# Review: Compliance standards for dairy cattle welfare in European countries

**DOI:** 10.1016/j.vas.2026.100587

**Published:** 2026-01-30

**Authors:** Letizia Debertolis, Louis Holighaus, Matthias Gauly, Thomas Zanon

**Affiliations:** Faculty of Agricultural, Environmental and Food Sciences, Free University of Bolzano, Piazza Università 5, 39100, Bolzano, Italy

**Keywords:** Dairy cattle, Animal welfare, Standards, Alpine region, Certification schemes

## Abstract

Over the last half century, animal welfare in livestock farming, particularly dairy cattle farming, has received increasing attention. Animal welfare legislation, as well as private and state-regulated standards and frameworks concerning animal welfare in dairy farming, has flourished globally, particularly in Europe. This study uses a modified systematic review approach to provide an overview of the various compliance frameworks (CFs) relating to dairy cattle welfare across Europe. These are classified according to the type of approach in: qualitative, or those that require only the achievement of a certain level of welfare; progressive, or those that encourage the constant improvement of welfare; disruptive, or those that propose a breakthrough in the management system, geared towards promoting welfare; and health monitoring programmes, considered separately. A qualitative assessment is conducted on nine selected CFs concerning the welfare of dairy cattle in five Alpine countries (France, Italy, Germany, Austria and Switzerland). Their different characteristics (organisation type and approach, interface with other relevant databases, covered subjects, and other qualitative features) are compared and presented in tabular form. This analysis reveals further differences between the CFs that transcend the type of approach adopted. Each country employs a personalised strategy. One distinction between the CFs is the presence of regulatory possibilities to integrate or compensate for shortcomings in certain areas, such as the absence of elements that promote welfare as regulated by the standard, with other virtuous practices. Furthermore, the comparison shows that health monitoring programmes are provided for in all states, thus underlining their continuing relevance.

## Highlights

• Over the past fifty years, animal welfare regulations in dairy farming have undergone significant changes worldwide. Each country has adapted its strategies to local needs.
• Large-scale regulatory and certification systems are now widespread but can sometimes be overly restrictive, creating difficulties for traditional small-scale dairy farms in mountainous regions such as the Alps and elsewhere. These farms may find it difficult to meet stringent standards due to structural, economic and environmental constraints, risking exclusion and potential harm to animal welfare. At the same time, it is essential to recognise the cultural and economic value of small traditional farms from a sustainability perspective.
• To address these issues, future regulations or amendments to existing ones should adopt flexible or inclusive approaches, such as compensatory measures, and balance ethical considerations, resource management and local conditions.
• Health monitoring programmes, whether stand-alone or integrated into general welfare policies, should give absolute priority to accurate and standardised diagnosis. This allows for the aggregation of data across countries and supports regions with small farms and local breeds, ultimately strengthening animal welfare outcomes.

## Graphical abstract

**Figure:**
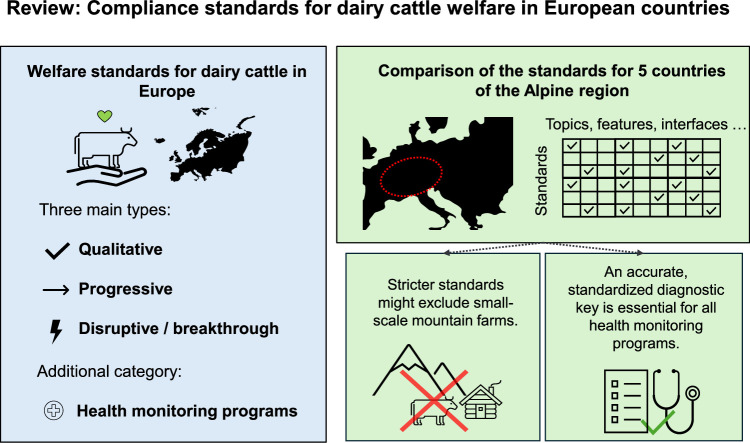

## 1 Introduction and scope of the review

Since Ruth Harrison's Animal Machines in 1964, which addressed the poor conditions of livestock, the welfare of farm animals has received growing attention, and farming practices that promote animal welfare have been increasingly favoured. (Alonso et al., 2020; Broom, 2011; Buller et al., 2018; Harrison, 2013). Consumers seem to prefer products that support animal welfare as they perceive them as healthier, tastier, cleaner, more acceptable, more environmentally friendly and more traditional (Blokhuis et al., 2008; de Boyer Des Roches et al., 2020; Graaf et al., 2016; Martelli, 2009). The desire for animal-friendly farming systems is also influenced by the concept of the Natural Living Frame. This is based on the idea that animals thrive best in conditions that mimic their natural environment (Boogaard et al., 2011; FAWC, 2011; Schulze & Deimel, 2012).

Animal welfare is a multidimensional concept with scientific, ethical, economic and political dimensions (Broom, 2011; Cornish et al., 2016; Hemsworth et al., 2015; Lund et al., 2006). Its focus lies on describing how well domestic animals can cope with their living conditions. Ensuring animal welfare means preventing health problems and psychological and emotional distress caused by housing conditions and management practices (WOAH, 2012). Specific indicators were developed to objectively assess livestock welfare (Aerts et al., 2006; Keeling, 2019). Over the years, research defined these indicators and classified them as resource- and management-based indicators (MBIs) on one hand, and animal-based indicators (ABIs) on the other hand (Bergschmidt et al., 2019; Keeling, 2019). MBIs provide information on the structure and management of the environment in which animals live, i.e. housing, available space, bedding, feeding, resting, water supply, access to pasture, milking practices and hygiene, pain management during medical procedures such as dehorning and castration, etc. This group of indicators is relatively easy to measure. They are considered indirect indicators because they do not provide direct information on welfare status, but only on factors that may influence it (Bergschmidt, 2017; Bergschmidt et al., 2019; EFSA, 2012). ABIs, on the other hand, provide an indication of animal welfare directly at the animal level. Aspects such as nutritional status, coat cleanliness, disease prevalence (mastitis, lameness), injuries, and behaviour are assessed directly by observing the animal. Besides these classic ABIs, based on clinical aspects, other validated animal-specific parameters can theoretically serve as health or welfare indicators but require laboratory testing or specialised instruments. They are less immediate than clinical signs, thus their routine use as indicators is not consistent (Vesel et al., 2020). These include haematological and biochemical blood profiles, comprising acute phase proteins (Loi et al., 2021; Tothova et al., 2016); blood-based metabolic indicators like beta-hydroxybutyrate and non-esterified fatty acids (Overton et al., 2017), milk immunological biomarkers, particularly haptoglobin, lactoferrin, and vascular endothelial growth factor (Zoldan et al., 2017); and cortisol measurements in hair and faeces for stress evaluation (Tallo-Parra et al., 2015). ABIs are generally more labour-intensive to measure. However, they are considered direct indicators and the most appropriate tools for evaluating animal welfare because they reflect the effects of MBIs (Brinkmann et al., 2016; Mondon et al., 2017). A further factor that has been identified as important in determining animal welfare is stockmanship attitude towards their livestock (Boivin et al., 2003; Losada-Espinosa et al., 2020; Platto et al., 2020).

To address the need to systematically define, understand and assess animal welfare, reference frameworks have been developed that introduce fundamental components or dimensions of welfare and provide a coherent structure for its assessment. Based on Soley (2025), the two most widely recognised general models for assessing welfare are the Five Freedoms and the Five Domains. The first is a set of recommendations organised into five basic animal needs called the “Five Freedoms” (freedom from thirst, hunger, and malnutrition; freedom from discomfort; freedom from pain, injury, or disease; freedom to express normal behaviour; and freedom from fear and distress). These were formulated in 1979 by the Farm Animal Welfare Council (FAWC, 2011) and remain a benchmark today. The second model was developed by Mellor and Reid (1994) and has been regularly reviewed and updated to incorporate developments in animal welfare science, with the latest update in 2020 (Mellor, 2016; Mellor et al., 2020). This model not only assesses the absence of factors essential to animal welfare, but also the presence of positive and constructive elements, and evaluates the balance between the two. Furthermore, due to increased public awareness, numerous initiatives dedicated to animal welfare in farming have also flourished in recent years, both at the private and state levels (Bock & Leeuwen, 2005; Veissier et al., 2008). In the European area, these initiatives range from regulatory frameworks established by the European Union (EU) and/or the national legislation of each country to voluntary certification schemes and industry-led guidelines that exceed the animal welfare standards in conventional livestock husbandry (Bock & Leeuwen, 2005; Veissier et al., 2008; Vogeler, 2019). Attendance in the voluntary programs is mostly tied to subsidies to motivate farmers. Products manufactured under higher animal welfare standards can be labelled (Bock & Leeuwen, 2005; Vogeler, 2019).

However, a proliferation of policies can lead to mistrust among stakeholders and consumers through fragmentation and disillusionment (Fraser, 2003; Mondon et al., 2017). Furthermore, comprehensive supranational regulation of animal welfare standards is lacking, and at the same time, diversity in programmes is necessary to reflect local needs and conditions. This could lead to reduced visibility for some voluntary programmes. In this regard, reviews and comparative descriptions of international policies could help identify best practices and improve welfare standards by incorporating successful elements from different regions, thereby enhancing animal welfare. These observations reflect the purpose of this review. A general overview of relevant certification schemes, regulations, and policies related to the welfare of dairy cattle in Europe is provided. Furthermore, for the five major Alpine countries (France, Switzerland, Germany, Austria and Italy), the characteristics and implementation criteria of their most relevant welfare standards are compared in detail. These five countries were chosen because of their geographical proximity, similar cultural and agricultural traditions, significant similarities in the size, structure, and management of dairy farms in their mountain areas, and the importance of the dairy sector in those areas.

## 2 Methodology

### 2.1 Compliance frameworks: definition and search strategy

For simplicity, the term ‘compliance framework’ (CF) is used in this review to refer to any type of animal welfare regulation, standard, policy or certification and labelling scheme. The CF search strategy followed a mixed systematic procedure, based on an adaptation of the PRISMA 2020 guideline (Page et al., 2021), with a multi-source approach. The modified PRISMA 2020 Flow Diagram is reported as Supplementary Material. Search engines and legal and scientific databases were searched for CFs in the selected regions (geographical Europe, including the five largest Alpine countries), as well as for relevant articles and reviews. A Google desk search was conducted using the following search query: (animal health AND animal welfare) (labelling OR quality OR certification OR standard) (dairy cattle). The word ‘Europe’ was omitted from the search criteria so as not to risk that the research would give priority to results from EU countries alone. The first five result pages of this desk search were examined. The databases Web of Science, Scopus, PubMed, FAOLEX, Eur-Lex, CAB Direct, WOAH Database, EFSA, national databases (national ministries and agency websites), and Google Scholar were searched, with search inputs tailored to each database. In some cases, direct contact was made with relevant authorities in the selected countries, like government agencies responsible for animal welfare, academic departments of animal science, and government-affiliated research and diagnostic institutes, to obtain additional information. The research revealed a snowball effect, in which certain CFs emerged from citations in papers or from other CFs.

### 2.2 Selection, screening, and prioritisation of CFs

The screening and prioritisation process is reported in the modified PRISMA 2020 Flow diagram (see Supplementary Material). The initial research resulted in 116 CFs, which were catalogued in an Excel table. After excluding CFs closed by the end of 2014, removing duplicates and other errors in the cataloguing, the number of CFs was reduced to 94. The first screening process eliminated 17 CFs because only CFs from Europe could be included. Furthermore, in this first step, 9 CFs that did not address dairy cattle as their subject matter were excluded. The following priority criteria were then identified and applied to the remaining 68 CFs:

• Accessibility and transparency: priority was given to CFs for which documentation is readily available online.
• Economic importance of the dairy sector in the country of origin: priority was given to CFs from countries where the dairy sector is most important.
• Importance of animal welfare in the area, based on tradition and previous policies: priority was given to CFs from countries with documented animal welfare policies.

The aim of this prioritisation was to further reduce the number of CFs to around ten for each of the two parts of this work (general overview and comparison of the five Alpine countries). Favouring the most accessible CFs and those likely to be applied on a larger scale, the criteria aligned with the aim of this review: to provide support in creating new policies or improving existing ones. Thus, eight CFs were selected as examples in the general overview, and nine were selected for comparison across the five Alpine countries. For the latter, since some standards are not unique, i.e. the requirements may vary depending on the situation or module chosen, a thematic comparison, examining only the topics covered by the standards and not the specifics, was used. The selected characteristics are presented in Table 1. The main characteristics of the nine CFs of the five Alpine countries have been summarised in tabular form to facilitate visual comparison (Tables 2, Table 3, Table 4).

**Table 1:** Characteristics and type of information or data retrieved from the selected standards.

	Type of information/data
Characteristics	
Country/region	Description
Organisation/property	Description
Label on products	Yes/no
Approach type	Description
Based on (protocol, standard)	Description
Contains prescriptions about	
Housing	Yes/no
Pasture	Yes/no
Milking	Yes/no
Calves	Yes/no
Transport	Yes/no
Exhibitions	Yes/no
Slaughtering	Yes/no
Feed	Yes/no
Water	Yes/no
Interface with	
Cattle registry	Yes/no
Slaughterhouse data	Yes/no
Electronic treatment registry	Yes/no
Collects data on	
Claw health	Yes/no
Udder health	Yes/no
Mortality	Yes/no
Other diagnoses	Yes/no

**Table 2:** Comparative and summary table of the fundamental structural characteristics of the nine selected compliance frames active in the Alpine countries.

	Country/Region	Organisation/property	Label on products	Main approach type	Based on (protocol, standard)	Sources
CF name						
CBPE/Boviwell	France	state	n.a.	qualitative	Own standard + Welfare Quality®	(CNE, 2023b; CNIEL, 2022)
Classyfarm	Italy	state	yes	qualitative	Welfare Quality®	(Bertocchi et al., 2025; Classyfarm, 2025)
Haltungsform	Germany	private	yes	progressive	own standard	(Haltungsform, 2024a, k, 2025)
ProGesund Bayern	Bavaria (Germany)	federal state (Bavaria)	no	h.m.p.	own standard (diagnosis code)	(Bayerische Landesanstalt für Landwirtschaft, 2025)
Gmon Baden Württemberg	Baden-Württenberg (Germany)	federal state (Baden-Württenberg)	no	h.m.p.	own standard (diagnosis code)	(Landesverband Baden-Württemberg für Leistungs- und Qualitätsprüfungen in der Tierzucht e.V., 2019, 2025)
Netzwerk Rindergesundheit	Switzerland	state	no	h.m.p.	own standard (diagnosis code)	(Arbeitsgemeinschaft Schweizerischer Rinderzüchter, 2017; 2020; Bundesamt für Lebensmittelsicherheit und Veterinärwesen, 2016, 2020)
Grüner Teppich	Switzerland	state	yes	progressive	own standard	(Branchenorganisation Milch, 2023a, 2023b, 2025)
AMA Gütesiegel	Austria	private	yes	qualitative	own standard	(AMA, 2022, 2024, 2025)
Gesundheitsmonitoring Rind	Bavaria (Germany)	federal state (Bavaria)	no	h.m.p.	own standard (diagnosis code)	(Bayerische Landesanstalt für Landwirtschaft, 2025)

Abbreviations: CF: compliance frames; h.m.p.: health monitoring program; n.a.: not available (this information could not be retrieved from the publicly available documentation).

**Table 3:** Comparative and summary table of the main animal welfare requirements integrated into the nine selected compliance frames active in the Alpine countries.

	Contains prescriptions about								
CF name	Housing	Pasture	Milking (hygiene and/or frequency)	Calves (housing and /or treatments)	Animal transport	Exhibitions	Slaughtering	Feed	Water
CBPE/Boviwell	yes	n.a.	yes	n.a.	n.a.	n.a.	n.a.	yes	yes
Classyfarm	yes	yes	yes	yes	no	no	yes	yes	yes
Haltungsform	yes	yes	no	yes (dehorning)	no	no	no	yes	no
ProGesund Bayern^1^	no	no	no	no	no	no	no	no	no
Gmon Baden Württemberg^1^	no	no	no	no	no	no	no	no	no
Netzwerk Rindergesundheit^1^	no	no	no	no	no	no	no	no	no
Grüner Teppich	yes	yes	yes	yes	no	yes	yes	yes	no
AMA Gütesiegel	yes	yes	yes	yes	no	no	no	yes	yes
Gesundheitsmonitoring Rind^1^	no	no	no	no	no	no	no	no	no

Abbreviations: CF: compliance frames; n.a.: not available (this information could not be retrieved from the publicly available documentation).

Sources: see Table 2.

1 Being health monitoring programmes, these CFs per definition do not contain prescriptions.

**Table 4:** Comparative and summary table of the main interfaces, diagnostic and animal-based inputs of the nine selected compliance frames active in the Alpine countries.

	Interface with			Input data collected			
CF name	Cattle registry (traceability and/or genetic improvement)	Slaughterhouse data	Electronic treatment registry	Claw health	Udder health	Mortality	Other diagnoses
CBPE/Boviwell	yes	n.a.	yes	yes	yes	yes	yes
Classyfarm	yes	yes	yes	yes	yes	yes	yes
Haltungsform	n.a.	yes	yes	no	no	no	no
ProGesund Bayern	yes	yes	yes	yes	yes	yes	yes
Gmon Baden Württemberg	yes	yes	yes	yes	yes	yes	yes
Netzwerk Rindergesundheit	yes	yes	yes	yes	yes	yes	yes
Grüner Teppich	yes	n.a.	n.a. (optimization of antibiotic use is mentioned)	no	no	no	no
AMA Gütesiegel	yes	yes	yes	no	yes	yes	yes (only QPlus)
Gesundheitsmonitoring Rind	yes	yes	yes	yes	yes	yes	yes

Abbreviations: CF: compliance frames; n.a.: not available (this information could not be retrieved from the publicly available documentation).

Sources: see Table 2.

## 3 Overview of the different types of dairy cattle welfare CFs in Europe with examples

Of the 50 continental European countries, 27 are members of the European Union (EU). Therefore, a summary of the EU’s legislation, supranational CFs, and guidelines concerning the welfare of dairy cattle is provided below. EU legislation on dairy cattle welfare is mainly governed by Directive 98/58/EC, which provides general protection for all farm animals but lacks specific standards for adult dairy cows. Only dairy calves receive targeted protection under Directive 2008/119/EU, which encourages group housing and natural behaviours. Transport and slaughter are regulated by Regulation 1/2005 and Regulation 1099/2009. Organic farming, which promotes natural processes and responsible resource use, also aims to achieve high standards of animal welfare and is regulated at the EU level. Since 1991, organic production has been governed by Regulation (EEC) No 2092/91, updated by Regulation (EU) 2018/848 in 2022. This new regulation strengthens controls and supports small farmers, but its definitions of animal welfare remain broad and allow exceptions. Eco-schemes, part of the Common Agricultural Policy (CAP) 2023–27, support farmers in adopting environmentally friendly and sustainable practices, including direct improvements in animal welfare such as better housing conditions. The European Food Safety Authority (EFSA) has published key documents and scientific opinions on dairy cattle welfare, including a report on the link between animal welfare and food safety (EFSA, 2009) and a comprehensive opinion with recommendations for improvement (EFSA, 2023). Finally, the Welfare Quality® Project, funded by the EU, has developed a comprehensive framework for assessing and improving farm animal welfare. This project resulted in a detailed, evidence-based tool to enhance animal welfare (Keeling et al., 2013).

It is possible to classify the different CFs by various criteria, such as time frame, labelling type, production line, and mandatory status. We adopted the classification system used by de Boyer des Roches et al. (2020), similar to that formulated in one of the Welfare Quality® project’s reports (Bock & Leeuwen, 2005), which outlines CFs with a qualitative, progressive, and breakthrough (or disruptive, or revolutionary) approach. Below, we illustrate the three categories with examples from across Europe. A fourth category, conceptually like qualitative CFs but in some ways overlapping all three, is represented by health monitoring programmes, described separately.

### 3.1 Qualitative approach

Qualitative CFs (quality marks) ensure compliance with animal protection laws and focus on traceability. While they often meet only minimum welfare standards, some go beyond regulations, covering aspects such as health, welfare, and environmental impact, and may include best-practice guidelines. Compliance is checked through audits or self-assessment. Traceability efforts can support the ideal of authenticity and, sometimes directly or indirectly, benefit animal welfare, especially when health monitoring, such as reporting diagnoses and antibiotic use, is required. Examples of qualitative approach CFs include:

#### 3.1.1 Red Tractor Assurance (United Kingdom)

The Red Tractor is a quality certification label managed by Assured Food Standards, a UK not-for-profit founded in 2000. It sets standards for various food production sectors, including poultry, dairy, beef, lamb, swine, crops, and produce, and involves over 46,000 farmers and 900 operators. Products can only display the Red Tractor label if they comply with requirements for production, transport, storage, and packaging, as verified by more than 350 independent inspectors through 60,000 assessments annually (Red Tractor, 2025a). In the dairy sector, over 11,000 farmers—95 % of UK milk producers—are certified under Red Tractor standards for housing, feeding, health, welfare, biosecurity, and transport (Red Tractor, 2025b). However, these standards do not go beyond legal animal welfare requirements or promote additional welfare practices, serving mainly as a market entry requirement for large retailers.

#### 3.1.2 Origin Green (Ireland)

Origin Green is Ireland’s national food and drink sustainability program, managed by the state-owned enterprise Bord Bia. It brings together government, industry, and the supply chain to promote sustainable agriculture and meet consumer demand for ethically produced food. The program sets measurable environmental targets and includes over 77,000 farms across sectors such as beef, dairy, poultry, eggs, and horticulture through voluntary Sustainability and Quality Assurance Schemes. Farms are independently audited every 18 months to verify compliance with standards covering greenhouse gas emissions, biodiversity, water use, energy, soil management, and socio-economic issues. Dairy farmers in Origin Green produce 95 % of Ireland’s milk. The scheme also covers processors and retailers, with farm-level accreditation under ISO:17065 and PAS 2050. Aligned farmers receive resources and must implement tailored sustainability plans. Audits confirm ongoing compliance, necessary for certification. While the emphasis is on environmental performance and efficiency, aspects of animal health and welfare are included, though access to pasture is not explicitly required. Companies benefit from improved efficiency, market access, and reputation for sustainable practices (Origin Green, 2023; 2025).

### 3.2 Progressive approach

Progressive CFs go beyond minimum animal welfare laws by introducing advanced practices. They may be inclusive—rewarding welfare improvements and progress—or exclusive, segmenting products by quality. These frameworks often rely on evaluation, labelling, and verification by independent or self-assessment bodies. Their main aim is the continual improvement of agricultural practices. Examples of progressive CFs include:

#### 3.2.1 Better Animal Welfare (Denmark)

The "Better Animal Welfare" label (Danish: “Bedre Dyrevelfærd”), introduced in 2017, encourages higher standards of animal welfare for livestock by offering producers a market-based incentive. Developed by the Danish Veterinary and Food Administration with input from industry stakeholders, this voluntary label exceeds European and Danish legal requirements. It applies to pork, chicken, veal, beef, milk, and dairy products, and is easily identifiable to consumers seeking higher welfare standards in products. The scheme involves no subsidies; participating farmers receive training and regular inspections by independent certifiers. Legislation also restricts calf slaughter and addresses on-farm mortality (Danish Veterinary and Food Administration, 2025).

#### 3.2.2 RPCSA Assured (United Kingdom)

The RSPCA Assured program, managed by the Royal Society for the Prevention of Cruelty to Animals (RSPCA), is a not-for-profit animal welfare certification scheme. Its standards, based on ongoing research and practical experience, aim to improve animal welfare throughout an animal’s life, from birth to slaughter (RSPCA, 2024a). For dairy cattle, the program requires clean, comfortable living conditions, access to pasture when possible, regular veterinary care, and opportunities for natural behaviours like grazing and socialising (RSPCA, 2024b). Compliance is verified through independent inspections, including unannounced audits. The program also provides farmer training and support (RSPCA, 2024a). Products carrying the RSPCA Assured label can reach higher-value markets, often attracting premiums due to the assurance of high welfare standards. This label helps consumers make informed choices and supports improved animal welfare by driving demand for responsibly produced goods (RSPCA, 2024b).

#### 3.2.3 Beter Leven (The Netherlands)

The Beter Leven (Better Life) scheme, established in 2007 by the Dutch Society for the Protection of Animals, is the leading and fastest-growing animal welfare certification program in the Netherlands. This non-profit initiative collaborates with NGOs, government, farmers, processors, retailers, and consultants to set and enforce standards that exceed legal requirements, while educating consumers about animal welfare through clear product labelling.

Beter Leven rates farms and products on a scale of 1 to 3 based on criteria such as living space, outdoor access, feed quality, and environmental enrichment. One-star farms provide improved conditions over conventional farming; two-star farms offer more space, outdoor access, and enriched environments; three-star farms meet organic-level standards, including maximum space and natural living conditions. Compliance is ensured through annual audits and unannounced inspections by both the Beter Leven Label Foundation and independent certification bodies, with additional oversight provided by third-party shadow audits and back-tracking checks.

The scheme covers various farm animals—including dairy cattle, laying hens, broiler chickens, pigs, beef cattle, calves, turkeys, and rabbits—and has significantly improved animal welfare and influenced consumer behaviour in the Netherlands. For dairy cows, protocols specify resting space, pasture access, nutrition, health care, and the prohibition of painful procedures across the star levels, with higher ratings requiring stricter standards and greater promotion of natural behaviours and preventive care (Dierenbescherming, 2019, 2022a, 2022b, 2022c, 2024).

#### 3.2.4 Weidemelk (The Netherlands)

The Netherlands, Ireland, and Scandinavian countries commonly use pasture grazing for dairy cattle. Stichting Weidegang launched the Weidemelk initiative in 2012 to promote pasture-based dairy in the Netherlands. While animal welfare is not explicitly stated, outdoor grazing is prioritised. Weidemelk certification requires cows to graze either a minimum of 2500 h over 120 days (max 3 cows/hectare) or at least 1500 h over 120 days (max 5 cows/hectare), with a minimum of 2 grazing hours between 6 AM and 10 PM. The certification reviews further management aspects, such as manure handling and compliance with EU regulations (i.e., interface with Eco-schemes), and offers farmer training and support. Several other European countries also produce or sell milk under the Weidemelk label (Stichting Weidegang, 2022).

#### 3.2.5 Svenskt Sigill (Sweden)

Svenskt Sigill is a Swedish private quality label indicating high standards in agriculture, covering animal welfare, environmental care, and food safety. Managed by Sigill Kvalitetssystem AB, part of the Federation of Swedish Farmers (LRF), the label relies on collaboratively developed standards that often exceed Swedish and EU laws. Certification involves initial farm assessments and ongoing inspections, requiring thorough record-keeping for traceability. Specific rules for dairy cattle mandate spacious living conditions, outdoor access, balanced diets, minimal antibiotic use, and humane handling, with stricter guidelines than legal minimums. The scheme includes different levels: Svenskt Sigill Basis, which assures compliance beyond standard regulations, and Svenskt Sigill Klimatcertifierad, which adds requirements for sustainability and reduced greenhouse gas emissions. Recognised as a trusted symbol by Swedish consumers, the label supports market access and premium pricing for certified products. Overall, Svenskt Sigill promotes sustainable, ethical, and high-quality agricultural production in Sweden (Svenskt Sigill, 2023)

### 3.3 Breakthrough approach

Breakthrough, or disruptive, CFs propose a radical change from the conventional system. The aim is to adopt alternatives that, over time, become the new standard for all production. This type of approach can be funded through public aid or the creation of official or private labels and aims to significantly improve animal welfare and product transparency, focusing on results rather than specific means. In this regard, any kind of organic certification is a disruptive approach. One particularly relevant example of the breakthrough approach is the KRAV label.

#### 3.3.1 KRAV (Sweden)

Sweden’s KRAV label represents a disruptive approach, aiming to significantly transform the farming system by promoting organic and biodynamic farming practices. This label supports extensive outdoor access for animals, the use of organic feed, and a prohibition on routine antibiotic use. The goal is to create a more sustainable and ethical farming system that sets a new benchmark for animal welfare (KRAV, 2024).

### 3.4 Health monitoring programs

**Fig. 1:**
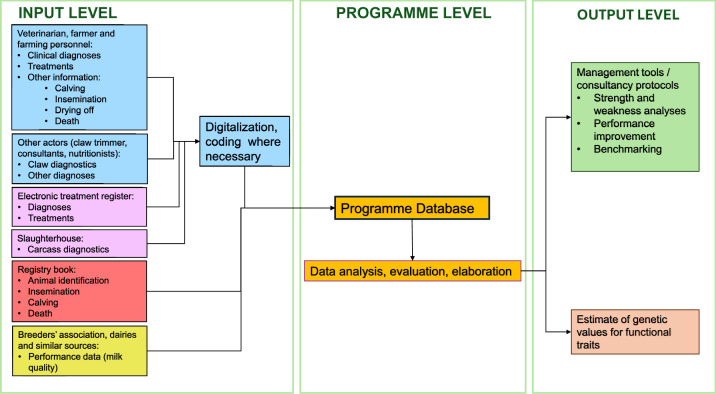
Schematic representation of the functioning of health monitoring programmes. These programmes usually consist of three levels: inputs, programme and outputs. Inputs are diagnoses and other information (e.g. from registries or performance data). The programme's core is a database. Outputs are consultancy protocols for improving farm management and health, and/or genetic value estimates. See text for more details.

Health monitoring programmes do not directly certify animal welfare and generally avoid classic Animal-Based (ABIs) and Management-Based Indicators (MBIs), instead using diagnostic health data. These initiatives may stand alone or be part of wider welfare frameworks, sometimes later integrated into larger systems. Data from registers and event documentation are combined and processed, mainly by breeders' associations to maintain animal genealogic data and implement their genetic improvement programmes, or by governments and veterinary health organisations for disease eradication (FAO, 2016). Initially, those programs were voluntary or limited to high-risk areas. However, major outbreaks like BSE and FMD in the early 2000s triggered significant losses and hampered market access (FAO, 2016). In response, the European Commission's Animal Health Strategy (2007–2013) emphasised prevention, making comprehensive health data recording essential for disease control. Many countries now require full traceability of animals and their products (FAO, 2016). Advances in mobile and web technologies have improved surveillance for human, animal and plant health. Traditional epidemiological surveillance relies on local expert monitoring, records and lab tests (McNeil et al., 2025; Smolinski, 2016). Instead, participatory surveillance, which gathers data directly from the public - particularly where conventional methods are impractical -has improved efficiency worldwide (McNeil et al., 2022, 2025; Smolinski, 2016). In Europe, advanced veterinary systems use professional participation for ongoing monitoring and evidence-based recommendations, though typically focusing on genetics or productivity rather than epidemiological surveillance. Nordic countries led the way in using diagnostic cattle data for genetic selection, a practice now in Austria, Germany, Switzerland (Egger-Danner et al., 2012, 2015), France (Govignon-Gion et al., 2016; Leclerc et al., 2019), Spain (Pérez-Cabal & Charfeddine, 2013), and Italy (De Monte et al., 2020). Programmes usually follow International Committee for the Recording of Animals (ICAR) guidelines and have a three-level structure: input, programme and output, summarised in Fig. 1. Diagnoses, entered by various professionals, cover clinical findings and key life events, with interfaces to antibiotic usage and carcass data. Integrated data feeds into a database, producing two main outputs: qualitative farm management analyses and genetic value estimates for functional traits.

## 4 Description and comparison of selected standards for the Alpine region

### 4.1 France

#### 4.1.1 La Charte des bonnes pratiques d’Élevage

France, one of Europe's largest countries, has various socio-economic CFs to enhance animal health and welfare, supported by government, semi-government, and private organisations. All three main approach types—qualitative, progressive, and breakthrough—are widely used. For dairy cattle, the primary framework is the Charte des bonnes pratiques d’Élevage (Engl.: Charter of good farming practices; CBPE), established in 1999 after BSE concerns to ensure traceability and animal health. The CBPE has since evolved into a comprehensive qualitative standard promoting sustainable livestock farming, with voluntary participation. Its criteria cover seven pillars: traceability, health, nutrition, milk hygiene, social sustainability, environment, and animal welfare. Animal welfare assessment was strengthened by introducing the mandatory Boviwell tool in 2022 as part of the ‘France Terre de Lait’ initiative, aligning dairy production with societal demands for welfare and sustainability (CNE, 2019; CNIEL, 2022).

#### 4.1.2 Boviwell

Boviwell, introduced in 2022, is a herd welfare assessment tool derived from the Welfare Quality® protocol, developed by Idele (Institut de l'Élevage, the French Livestock Institute) and Cniel (Centre National Interprofessionnel de l’Économie Laitière, the French Interprofessional Centre for the Dairy Economy) and regulated at State level. The in-person audit, performed every three years, evaluates dairy cattle welfare based on the five fundamental freedoms, covering 20 features of farm management. Boviwell uses both Animal-Based (ABIs) and Management-Based Indicators (MBIs), favouring ABIs, and assesses aspects such as body condition, access to food and water, pens bedding quality, health, injury rates, and freedom to express natural behaviours. The scoring system combines thresholds, benchmark deviations, and a hierarchical flow chart to ensure comprehensive evaluation without compensating poor results in one area with good results in another. Outcomes are summarised in graphs and classified into four levels from “Excellent” to “Not Classified.” Specially trained consultants conduct the assessments, and an independent body certifies compliance. Farmers are encouraged to improve their scores and achieve excellence (CNE, 2023a, 2023b; Mounaix et al., 2021). Thus, Boviwell, as a standalone tool, represents a progressive approach.

### 4.2 Italy

#### 4.2.1 Classyfarm

Italy has created a centralised animal health monitoring system called Classyfarm. Developed by the Ministry of Health, the Istituto Zooprofilattico Sperimentale di Lombardia ed Emilia Romagna (one of the Italian veterinary diagnostic and research institutes affiliated with the National Health Service), and the University of Parma, Classyfarm began in pig farming in 2019 and expanded to other sectors, including dairy in 2023. It integrates farm audit data with slaughterhouse, livestock register, and treatment records, giving comprehensive insights for each farm. The system assesses dairy farms on biosecurity, management, personnel, housing, animal-based indicators, and risk factors, with protocols for different housing systems. Annual assessments are performed by veterinarians or trained certifiers, who also guide farm managers, and independent inspections occur every three years. Classyfarm aims to enhance animal welfare and health, prevent disease, boost food safety, and limit antimicrobial use. Participation is voluntary but required for the SQNBA label, which links to CAP financial support and appears on consumer dairy products (Bertocchi et al., 2025; Classyfarm, 2025).

### 4.3 Germany

#### 4.3.1 Haltungsform

The Haltungsform initiative in Germany offers a clear system for labelling animal-based food products by their welfare standards (Haltungsform, 2024b). Launched in 2019 as a voluntary, private effort, it began with meat products and expanded to dairy by 2022 (Haltungsform, 2024b, 2025). Originally consisting of four levels, the top tier was split in 2024, creating a fifth level for organic programs (Haltungsform, 2024a). Independent agencies check compliance.

For dairy cattle, the Haltungsform standard encompasses six categories: living space, type of housing, management of calves’ dehorning, environmental enrichment, feed, and health monitoring. Level 1 meets legal minimums: cows are kept indoors, each has a cubicle, QS-certified feed (Qualität und Sicherheit, Engl.: Quality and Safety, the leading private food quality assurance system in Germany) is used, and basic veterinary care is provided, with no enrichment or pasture. Tie stalls are allowed. Level 2 improves indoor conditions, allowing more space and limited outdoor access; enrichment like brushes is included, but most requirements mirror Level 1. Tie stalls require some pasture access. Level 3 bans tie stalls, mandates more space, outdoor access, GMO-free feed, compulsory enrichment, and enhanced health checks. Level 4 increases space, mandates constant outdoor access, organic or sustainable feed, frequent vet visits, and restricts dehorning to exceptional cases. Level 5 covers organic farms, similar to Level 4 but requiring fully organic feed and complying with EU regulations (Haltungsform, 2024a, 2024b, 2025).

By distinguishing these levels, Haltungsform helps consumers choose products based on animal welfare standards, encouraging better housing for dairy cows through informed purchasing. Thus, Haltungsform is a typical progressive CF.

#### 4.3.2 GMON Baden-Württemberg

In 2009, institutions in Baden-Württemberg, Germany launched GMON (Gesundheitsmonitoring Rind, Engl.: bovine health monitoring), a collaborative bovine health monitoring initiative. Data from regular performance tests was supplemented with health information provided by farmers and veterinarians to simplify farm management and enable comparison between herds. The project also aimed to identify genetic values for key diseases, supporting the breeding of healthier cattle. By January 2021, over 1400 companies and 141 veterinary practices had participated, allowing for genetic evaluations, especially for Fleckvieh, Braunvieh, and Holstein breeds. In 2016, the Klauencheck BW project began to track hoof health through data from farmers and claw trimmers. Around 30,000 individual diagnoses were registered using ICAR codes. Data interfaces were created between the recording programmes and the computer system of the Landeskontrollverband (LKV, a technical and administrative dairy service control association operating at the regional level in Germany and other German-speaking countries). Since the Klauencheck BW project conclusion in 2019, its diagnoses are integrated into GMON (Landesverband Baden-Württemberg für Leistungs- und Qualitätsprüfungen in der Tierzucht e.V., 2019, 2025).

#### 4.3.3 ProGesund Bayern

The ProGesund project (2009–2019), led by the Bavarian Institute for Agriculture and funded by the Bavarian Ministry of Food, Agriculture and Forestry, aimed to improve herd health management on dairy farms, support veterinarians, and estimate genetic values for breeding bull health. It integrated diagnostic data from farmers, veterinarians, agencies such as Tiergesundheitsdienst Bayern e.V. (Bavarian Veterinary Health Service) and Fleischprüfring (Meat Control Circle), insemination technicians, and claw trimmers. Health results were analysed with milk yield and registry data to provide farmers with detailed evaluations on udder, fertility, metabolic, calf, and claw health. Genetic health assessments were conducted together with partners from Austria, Baden-Württemberg, North Rhine-Westphalia and Schleswig-Holstein. Since 2019, Pro Gesund has been incorporated into the LKV of Bavaria (Bayerische Landesanstalt für Landwirtschaft, 2025).

### 4.4 Switzerland

#### 4.4.1 Netzwerk Rindergesundheit

Switzerland's Netzwerk Rindergesundheit (Cattle Health Network) is a centralised system for monitoring animal health and traceability. Veterinarians and farmers insert diagnoses and treatments into the system via software, using their own standardised code. This program primarily collects data on bovine diseases and provides diagnostic benefits at both the individual farm and the regional level. It allows targeted action to be taken by identifying weaknesses and comparing them with those of other farms. As information on disease incidence is linked to genealogical information, via the herdbook, it is possible to analyse disease incidence in different lines and estimate heritability. Cattle breeding associations can use this data in breeding evaluations and promote disease-resistant lines through selective programs (Arbeitsgemeinschaft Schweizerischer Rinderzüchter, 2017; Bundesamt für Lebensmittelsicherheit und Veterinärwesen, 2016, 2020).

#### 4.4.2 Grüner Teppich

Grüner Teppich (Green Carpet) is the informal name for the Branchenstandard Nachhaltige Schweizer Milch, a set of programmes and standards aimed at improving practices in the Swiss livestock sector since 2019. Its focus is on environmental sustainability, but it also covers animal health and welfare. The policy is designed to be flexible, allowing farmers to select approaches that suit their needs. Compliance with Grüner Teppich requires meeting 10 basic requirements and two additional ones from a selectable list, while processors must demonstrate sustainability. Milk that meets these criteria receives a direct supplemental payment (Branchenorganisation Milch, 2025).

The 10 basic requirements (Branchenorganisation Milch, 2023a) include: fulfilment of the Ökologischer Leistungsnachweis (Proof of Ecological Performance), which covers fertiliser budgeting, biodiversity, crop rotation, soil and plant protection, and animal welfare, essentially requiring adherence to Swiss animal protection law; participation in at least one Confederation animal welfare programme (BTS, RAUS, or Weidebeitrag – see below); use of sustainable feed (especially certified soy); avoidance of palm oil and fat; optimised antibiotics use; minimum 21-day retention of calves on the birth farm; avoidance of slaughtering pregnant cows; compliance with guidelines of the Swiss Cattle Breeders' Association at exhibitions; milking at least twice daily with a maximum 14-hour interval during lactation; and registering a name for every cow in the Swiss tracking database. From the eight additional requirements, producers must select two: combining federal animal welfare programmes; achieving a minimum lifetime yield; no prophylactic antibiotics; use of complementary medicine; documented family worker remuneration; recognised training farm status; regular staff training; and hosting a school visit annually (Branchenorganisation Milch, 2023a).

##### 4.4.2.1 BTS, RAUS and Weidebeitrag

Animal welfare programmes in the Grüner Teppich encourage animal-friendly production through financial contributions. Three types of contributions are contemplated in this CF: BTS (for animal-friendly housing), RAUS (for regular outdoor access), and Weidebeitrag (for extensive grazing).

BTS requires stables to have separate areas for resting, moving, and feeding, allowing animals to freely switch activities. Resting areas must feature soft bedding, and loose housing is compulsory. RAUS mandates that animals spend time outdoors—either at pasture or in exercise areas—for 26 days per month in summer and 13 days per month in winter. Tethered housing is still permitted if outdoor access requirements are met. The Weidebeitrag program supports farms with high grazing levels by requiring cattle to be outside for 26 days per month in summer and 22 days per month in winter, while pastures must provide at least 70 % of daily dry matter during the grazing season. It is possible to combine BTS with either RAUS or Weidebeitrag, but RAUS and Weidebeitrag cannot be combined. (Branchenorganisation Milch, 2025).

##### 4.4.2.2 Compensation programs

Farms that do not meet the standard Grüner Teppich criteria, particularly the BTS criterion, have three compensation options: Basis Gesundheitsprogramm Milchvieh (basic health program), Sömmerung (summer grazing), and 8 Aren Wiesenfläche pro Kuh (8 ares of grassland per cow). In the basic health program, farms must complete an annual veterinary check and provide cows with open-air access at least 26 days per month in summer and 30 days in winter. The summer grazing option requires cows to spend at least 80 days per year on summer pastures, with open-air access on the valley farm for 26 days per month in summer and 13 days in winter. The grassland option mandates at least eight ares of meadow per cow for fresh feed, plus open-air access similar to the summer grazing scheme (Branchenorganisation Milch, 2025).

### 4.5 Austria

#### 4.5.1 AMA Gütesiegel

The AMA Gütesiegel (AMA Quality Seal) is managed by Agrarmarkt Austria (AMA) to certify the quality and traceability of Austrian agricultural products. Its CF is of qualitative type, ensuring that products bearing its quality label meet or exceed legal regulations in terms of quality and origin. Key features of the AMA Gütesiegel label are traceable Austrian origins, higher-than-legal quality standards, and independent verification. In addition to its basic version, which ensures compliance with legal requirements, especially in terms of dairy farming, the AMA Gütesiegel offers several facultative modules (Freiwillige Module) designed to enhance specific quality aspects or product characteristics:

Tierhaltung plus (Animal Husbandry Plus) is based on the AMA Quality Seal Basic Guideline and requires independent, unannounced checks. Mandatory elements are veterinary care, udder health checks, participation in animal health programs, GMO-free European feed, enriched environments, welfare-enhancing housing (loose housing in an open-front stable or year-round outdoor area), and regular pasture or outdoor access for dairy cows.

Heumilch (Haymilk) promotes traditional hay-based dairy farming with at least 75 % fresh and/or local hay feed; silage is not permitted.

Almmilch/Alpmilch (Alpine Milk Farming) supports on-site milk production in alpine pastures, requiring set grazing periods, GMO-free feed, and natural/supplementary feeding without silage or fermented hay.

Qplus-Kuh monitors cow metabolism during calving with the ‘KetoMir’ method, which checks milk for early ketosis signs and metabolic issues. An early warning system uses indicators like rearing losses and stillbirths for ongoing assessment, enabling preventive health measures for cows and calves.

Gentechnikfreie Fütterung (Gene Technology Free Feeding) ensures that the entire feed production chain is GMO-free.

Bergerzeugnis (Mountain Product) certifies milk from animals raised mainly in mountain areas, with most feed sourced locally and processing usually within 30 km of these areas (AMA, 2022, 2024).

#### 4.5.2 Gesundheitsmonitoring Rind

Since mid-2006, Austria has developed a central cattle health monitoring system called Gesundheitsmonitoring Rind (Cattle Health Monitoring), in collaboration between breeders, testing organisations, veterinary services, research groups and ministries. Initially a short-term project, its methods and data infrastructure have been integrated since 2010 into the routine operations of the Tiergesundheitsdienst (TGD), an animal health service organization regulated by the state (KVG, 2025). The programme aims to collect diagnostic data for herd management and breeding. Veterinarians record diagnoses using a special 2-digit code on medication records, which interface with the official medication registry. With consent, key data (animal identity, farm and vet numbers, diagnosis date and details) are stored in the cattle database (Rinderdatenverbund, RDV). Data may be entered during milk yield tests, sent electronically by vets, or added directly by farmers. This system enables monitoring herd health, early problem detection, and benchmarking, while the integration of databases and herd books with the animal health service allows calculation of breeding values and continual improvements in management and animal welfare (Oberösterreicher Tiergesundheitsdienst, 2025; Rinderzucht Austria, 2025a).

#### 4.5.3 Klauen-Q-Wohl

The Klauen-Q-Wohl (Claw Health) project began in 2017 to improve claw health across Austria (Rinderzucht Austria, 2018). By 2016, claw and limb diseases caused about 8 % of culling cases. Both animal welfare and financial losses motivated efforts to enhance hoof health (Egger-Danner & Kalcher, 2017; Rinderzucht Austria, 2018). From October 2017 to December 2020, the project established an electronic system for recording hoof care and lameness data, helping farmers and trimmers monitor herd hoof health and act early. The system also supported better herd management and breeding decisions. Data collection involved hoof trimmers, farmers, and veterinarians, enabling comparisons and tailored recommendations. As a result, genetic values for claw health were identified. After the project, the ‘Klauenprofi’ app was launched for LKV members, allowing easy documentation and direct reporting of hoof care and issues via their national cattle register login. Users can enter data, view reports, and perform detailed analyses with reference values.

## 5 Qualitative comparison of the standards of the Alpine area

The comparison focused on the content of the CFs rather than the levels of compliance and is presented visually in the form of tables (Tables 2, Table 3, Table 4). For the comparison, CBPE and Boviwell were considered together as the welfare assessment of CBPE is performed using Boviwell. Furthermore, Klauen-Q-Wohl, being a completed project, was not included as a separate CF in the comparison.

### 5.1 Fundamental structural characteristics

Table 2 presents a comparative analysis of CFs in terms of their fundamental structural characteristics. Clear similarities can be observed between CFs with the same type of approach. The health monitoring programmes we considered (ProGesund Bayern, Gmon Baden Württemberg, Netzwerk Rindergesundheit, Gesundheitsmonitoring Rind) are almost completely overlapping. They are generally based on their own standard or diagnostic code (as mentioned, generally based on ICAR), are managed at state or federal state level, and do not use product labelling. The CFs in the strictest sense, i.e. those focused fully or partly on animal welfare and compliance with standards, mostly incorporate direct assessment tools into product labelling (Classyfarm, Haltungsform, Grüner Teppich and AMA Gütesiegel) as an interface with consumers. Only in the case of CBPE/Boviwell was it not possible to find any indication of labelling.

### 5.2 Main animal welfare requirements

Table 3 provides a comparative overview of the main animal welfare requirements integrated into each CF in the Alpine region. It shows that welfare-specific CFs (CBPE/Boviwell, Classyfarm, Haltungsform, Grüner Teppich and AMA Gütesiegel) extensively integrate resource-based criteria into their compliance requirements, focusing largely on environmental and management factors. In the case of health monitoring programmes, however, none of the CFs assessed contain such requirements, as factors that may influence animal welfare are addressed indirectly through the systematic monitoring of health data and performance metrics in those. None of the nine CFs considered contain requirements regarding animal transport. One CF (Grüner Teppich) explicitly addresses animal welfare during exhibitions. Two CFs (Grüner Teppich and Classyfarm) specifically mention criteria for slaughter. Finally, three CFs (CBPE/Boviwell, Classyfarm and AMA Gütesiegel) provide explicit requirements on water supply.

### 5.3 Interfaces, diagnostic and animal-based inputs

Table 4 summarises the main interfaces, as well as the main diagnostic and animal-based inputs for the different CFs. As regards interfaces, apart from a few cases where information is not entirely available in freely accessible documents (interface with cattle registry for Haltungsform, interface with slaughterhouse data for CBPE/Boviwell and Grüner Teppich, and interface with the treatment register for Grüner Teppich), all CFs considered use all three interfaces. Concerning diagnostic data, Haltungsform and Grüner Teppich do not collect any, as they are based on a system of environmental and management prescriptions. All other CFs considered collect the diagnostic categories in question.

## 6 Discussion and future perspectives

In the five selected countries, all belonging in part or entirely to the Alpine region, there are fundamental structural disparities in the organisation of animal welfare control systems. Each state has developed its own concept, and those differences prevent us from making a comparative assessment of quality that goes beyond a simple thematic comparison. In fact, the application of a framework specifically designed for the critical evaluation of standards and CFs, such as that proposed by More et al. (2017) was unfeasible, due to the lack of technical specificity of the free-access information available for some of the CFs selected, as well as the structural and conceptual differences between them. As mentioned, more comprehensive and generic animal welfare framework do exist: e.g., the Five Freedoms (FAWC, 1979), the Welfare Quality® (Botreau et al., 2009), the model proposed by Bartussek et al. (2000) or the Five Domains by Mellor et al. (2016; 2020). Using one of those would involve assuming some level of compliance for each CF and then comparing the results across countries in light of the chosen model. This or similar methods were successfully employed in analytic comparative studies (Annen et al., 2013; Lanzoni et al., 2023). In our case however, the assumption would not necessarily reflect the real situation in the chosen country, given our intention to focus on the common element, i.e. the Alpine region. In fact, the size of the Alpine region in relation to the total area of the country varies between the states involved, and European mountain farming characteristics differ considerably from the general standard (Cialdella et al., 2009; Marini et al., 2011; O’Rourke et al., 2016; Santini et al., 2013). Therefore, even if it is a simpler option, in the present case only a qualitative comparison is free from the risk of bias and allows observations and conclusions, presented in the following.

### 6.1 Inclusivity and exclusivity of standards. Impact on the socio-economic scenario of European dairy cattle farming

Observing the different criteria and strategies of the selected CFs, we can introduce a more theoretical evaluation above the proposed classification into qualitative, progressive and breakthrough approaches, defining inclusive and exclusive standards. Exclusive CFs are those with possibly broader standards, for which, however, no alternatives are allowed: the approach is all in or all out. Inclusive approaches start from a basic guideline with a few fixed points to be respected and then offer different variations and compensations to adapt to the situation. Considering this classification, Classyfarm is an example of exclusive approach, as is CBPE with Boviwell. Conceptually, all of the health monitoring programmes we considered (ProGesund Bayern, Gmon Baden Württemberg, Netzwerk Rindergesundheit in Switzerland and Gesundheitsmonitoring Rind in Austria) are also exclusive. However, this classification is not particularly relevant for this type of programme. Their focus is not specifically on improving welfare through various measures, but on improving health through intensive diagnostic monitoring. Therefore, it would not be useful to provide for compensatory variants, as the approach focuses by definition on a single factor. AMA Gütesiegel should be considered intermediate, as its basic structure is exclusive but extended by the presence of various modules to adapt to different situations and needs, which can also be combined, providing a certain degree of inclusivity. Another intermediate between exclusive and inclusive is Haltungsform, since the presence of five levels allows for a certain degree of inclusivity, even if only the highest levels are favoured by this CF’s marketing. The only example of a fully inclusive approach, within our selected CFs, is Grüner Teppich, with its extensive compensation possibilities.

Considering the situation of Europe, and particularly the Alpine region, we believe that inclusivity is a decidedly advantageous feature for an animal welfare standard and a crucial point to consider for improvement of current and/or creation of new policies. Livestock farming in Europe is a key element of the continent's cultural history, and its transformations have accompanied the evolution of agriculture hand in hand since its dawn (Gaillard et al., 2009; Tieskens et al., 2017). The co-evolution of livestock farming, agriculture and cultural heritage in Europe is evident when considering the cultural and territorial diversity that has existed on the continent throughout history and in the contemporary era. In fact, in Europe, and particularly in mountain areas such as the Alps, it is possible to identify distinct biocultural landscapes, i.e. areas where the interaction between human activities (farming, livestock breeding, fishing and cultural activities) and the natural environment has shaped the territory over time (Agnoletti & Rotherham, 2015; Baránková & Špulerová, 2023; Stepp et al., 2005). They have significant cultural value that is expressed in traditional knowledge, language and customs linked to the territory and often host high levels of biodiversity thanks to sustainable practices and a deep ecological knowledge, developed and adapted over millennia (Agnoletti & Rotherham, 2015; Baránková & Špulerová, 2023; Stepp et al., 2005). However, it should be emphasised that biocultural landscapes account for only a limited part of the European agricultural landscape. This is particularly true for dairy farming systems. In this context, situations vary greatly between countries and even within the same country (Díaz de Otálora et al., 2022). The intensification of European agriculture since the second half of the 20th century has led to the exploitation of natural resources and ecosystems, negatively impacting climate and biodiversity, and causing the globalisation and biocultural homogenisation, the contemporary loss of biological and cultural diversity (Rozzi et al., 2018). This has mainly affected the most fertile and accessible areas, such as the lowlands (Heindorf et al., 2025; Jongman, 2002). Nowadays, European dairy farming systems can be broadly divided into two types: larger, usually intensive, high-input farms, which are more common in flat areas, and smaller, more traditional, low-input farms in more isolated areas (Bijttebier et al., 2017; Díaz de Otálora et al., 2022; Scollan et al., 2017). The latter are type is frequent in the Alpine area, where livestock farming plays a major role in the regional economy. They are characterised by a high percentage of semi-natural vegetation than most agricultural land. There is little or no use of agrochemicals, and a tendency to maintain a range of 'traditional' management practices, which benefits biodiversity (Bijttebier et al., 2017; Díaz de Otálora et al., 2022; Scollan et al., 2017). Also, continuity in management practices guarantees many species periods of relative stability, which facilitates their adaptation to prevailing conditions (Baldock, 1999). There is also usually a prevalence of traditional local breeds (Edwards et al., 2011). Alpine low-input dairy farming is often linked to and sometimes coincides with biocultural landscapes. Any animal welfare standards applicable in the Alpine region must therefore be adaptable to this wide range of circumstances on individual farms, while obviously aiming to improve the welfare of the animals concerned. However, in the practical implementation of any large-scale animal welfare standard, especially national or regional, there is a risk of losing sight of the original purpose and attributing importance to organisational ease, practicality or even marketing strategies, an issue addressed by several authors (Aerts et al., 2006; Holighaus et al., 2023). Instead, what matters is to extend good practices to as many animals as possible, as soon as possible. This goal can probably be achieved more effectively if the policy adopts a flexible and inclusive approach, allowing farmers to offset any structural deficiencies with extensively virtuous practices in other areas. Conversely, exclusive standards offer less flexibility and may disadvantage small businesses, because of the impossibility of adequately addressing local conditions. In today's global context, it is also crucial to prioritise environmental sustainability in all sectors. However, this can be challenging to reconcile with the ethical considerations that demand urgent improvements in animal welfare in the short term. Inclusive approaches would minimise structural disruptions, thereby reducing resource waste while promoting adaptable and compensatory measures for improving animal welfare. Thus, these approaches might be more forward-looking, also from a sustainability point of view. In recent years, a change in perspective has emerged, prompting a reconsideration of the potential of the livestock sector in the context of the circular economy and biodiversity conservation. Biocultural landscapes have been increasingly recognised as fundamental to conservation efforts, as they can serve as efficient and virtuous models of sustainability (Heindorf et al., 2025). From this holistic socio-economic perspective, it is possible to consider the dairy sector not as a threat, but rather as a resource. This is particularly true for traditional livestock farming, with small local businesses that are rooted in the territory and are often involved in its conservation (Heindorf et al., 2025; Marini et al., 2011). In this regard, other factors play an important role. For example, the known risk of confusion caused by excessive labelling (Fraser, 2003; Mondon et al., 2017) and the constant price pressure on producers (Costa et al., 2022; Simões et al., 2025) are other issues that argue in favour of integrating compensation options into existing policies that do not provide those.

### 6.2 Animal health programmes: ongoing relevance, and challenges

Another point arising from the comparative assessment of the various CFs is the importance of animal health monitoring programmes. In fact, some type of health monitoring programme is in place in each of the five states we analysed. As already mentioned, the intensification of dairy farming since the second half of the 21st century has significantly improved productivity, becoming nowadays even more environmentally efficient in absolute terms than in post-war times, against the common perception (Capper et al., 2009). However, it has undeniably had negative consequences too. In addition to those already mentioned (unbalanced exploitation of environmental resources, increased pollution and carbon emissions with detrimental effects on the climate, standardisation of procedures with consequent loss of biocultural heritage), it has also had a demonstrated negative impact on animal health. The spread of so-called productive diseases, i.e., reproductive and metabolic disorders (Cainzos et al., 2022; Lucy, 2001, 2019), lameness (Dolecheck & Bewley, 2018), and mastitis (Hogeveen et al., 2019) has grown over the years. These diseases cause reduced production, increased mortality or premature slaughter of animals and therefore result in major annual losses for farms. Many authors have quantified these losses using various approaches, e.g. as reviewed by Steeneveld et al. (2024). However, direct economic losses due to production diseases are not the only consequences. The presence of disease and, therefore, some level of pain or discomfort is in itself an ethical problem, as it denies animals one of their four fundamental freedoms (FAWC, 1979). There is also the growing consumer awareness that has developed in recent years regarding animal welfare and health, antibiotic resistance and antibiotic residues in food of animal origin (e.g., Redding et al., 2021; Richter et al., 2025), which result in indirect economic losses due to shifted demand. These factors have moved the focus of breeding programmes from productivity traits alone to include functional and fitness traits such as longevity, robustness, good fertility, ease of calving and resistance to mastitis (Egger-Danner et al., 2015; Miglior et al., 2017; Pavón, 2013). Fitness traits in livestock breeding usually have very low heritability and in some cases have a negative genetic correlation with production traits (Egger-Danner et al., 2015; Heringstad et al., 2003; Pryce et al., 2016), which makes them particularly difficult to select for (Willam & Simianer, 2017). It is therefore necessary to collect health performance data meticulously and systematically. This can be done with a health monitoring system, which has the added advantage of helping farmers identify and correct weaknesses and deficiencies in their farms, veterinarians improve surveillance, and other mentioned benefits (Egger-Danner et al., 2012; Heringstad et al., 2003; Heringstad & Østerås, 2013; Zapf et al., 2015). In particular, health events recorded by farmers are a rich source of data for genetic improvement (Parker Gaddis et al., 2012; Pryce et al., 2016; Wenz & Giebel, 2012). It should also be noted that most of the phenotypic data collected over the years by various programmes has largely concerned modern, specialised breeds, primarily Holstein Friesian (Egger-Danner et al., 2015). However, as mentioned, local breeds, sometimes dual-purpose, are widely present in Alpine areas and are better adapted to local conditions (Edwards et al., 2011). Therefore, to ensure robust adaptability and mitigate the decline of favourable traits such as longevity and fertility, typical of those breeds, attention has also been turned to these traits (Egger-Danner et al., 2015). In this regard, it is important to consider the diagnostic precision in data collection by all sources. High-quality, precise phenotypic data are a crucial prerequisite for calculating population parameters, especially in populations with limited numbers, such as local breeds. If this precision is not given, for example, because the diagnostic key in the programme is formulated too generally, the data collected can only be used partially and are therefore partly inconclusive for genetic purposes. This has been demonstrated to be the case for the Italian system now included in Classyfarm (De Monte et al., 2020; Zanon et al., 2024). On the other hand, Germany and Austria have been successfully interfacing their diagnostic data for more than a decade because they use precise and compatible diagnostic keys. Since 2010, breeding values for health traits like mastitis, early fertility disorders, cysts, milk fever, as well as many more health and non-health traits, have been published as part of the joint project “Zuchtwertschatzung” (Engl.: breeding value estimation) between those countries. The genetic value estimate covers several dairy breeds, both internationally widespread, such as Holstein Friesian and Brown Swiss, and local breeds, including Fleckvieh, Tyrolean Grey and various beef and conservation breeds (Bayerische Landesanstalt für Landwirtschaft, 2025; Landesverband Baden-Württemberg für Leistungs- und Qualitätsprüfungen in der Tierzucht e.V., 2025; Rinderzucht Austria, 2025b). The exact diagnostic keys of the health monitoring programmes in our study were not available for all CFs. Therefore, we are unable to provide an evaluation of the accuracy, adequacy and ease of standardisation of their diagnostic keys. Nevertheless, it is evident that a diagnostic key with those qualities is an essential feature of a CF. Analogous to inclusivity, its introduction should be considered in CFs where it is not present and prioritised in the creation of new policies. In this regard, smart or precision farming technologies—such as accelerometers, cameras, ruminal boluses, biosensors, and acoustic devices—offer significant potential for integration into animal welfare CFs (Leliveld et al., 2024; Stygar et al., 2021). In fact, these sensor systems can automate data collection, reducing reliance on manual input in health monitoring programs, and allow farmers to self-monitor the welfare of their herds more continuously and accurately. Organisations could also employ sensor-derived data for independent welfare compliance assessments. Despite their promise, these technologies must undergo thorough external validation to ensure that their measurements are accurate and reliable, particularly for screening complex, multifactorial conditions such as mastitis (Mottram, 2016). Currently, only a small fraction of marketed sensors, mostly accelerometer-based, have been properly validated in commercial farming contexts (Borchers et al., 2016; Stygar et al., 2021).

The health monitoring programmes examined in this study were either stand-alone (Germany with programmes at the state-regional level, Austria and Switzerland at the state level) or integrated into broader frameworks (Italy and France, both at the state level). It is reasonable to assume that separate programmes with a single objective are easier to organise and therefore offer efficiency gains. Furthermore, due to their single purpose, it is presumably easier for stand-alone programmes to develop a diagnostic key with advanced characteristics of accuracy, adequacy and standardisation, and that can be interfaced with data from other countries if necessary. Indeed, as already mentioned, the stand-alone programmes in Germany and Austria have been able to combine their data, increasing the data pool and the accuracy of calculations for breeding programmes. However, these assumptions could be countered by the argument that the co-existence of several programmes in the same policy area may be counterproductive, creating confusion and decision-making difficulties for stakeholders and consumers (Ferguson et al., 2013; Fraser, 2003; Mondon et al., 2017). Health monitoring programmes integrated into broader CFs would avoid this problem. However, the data in our possession, based solely on qualitative assessment, are not sufficient to favour either hypothesis. A larger-scale comparison would be necessary, for example considering the data on adherence to the various types of CFs and the relative frequency of diseases.

## Ethics approval

This study did not require the approval of the Ethical Committee for the Care and Use of Experimental Animals of the Free University of Bolzano.

## Data and model availability statement

None of the data was deposited in an official repository.

## Declaration of generative AI and AI-assisted technologies in the writing process

No text-generating AI was used. AI-assisted technologies were used for translation (Deepl.com) and to check grammar and orthography (Grammarly.com). After using these tools, the authors reviewed and edited the content as needed and take full responsibility for the content of the published article.

## Ethical statement

Hereby I, Letizia Debertolis, consciously assure that for the manuscript “Review: Compliance standards for dairy cattle welfare in European countries” fully complies with Elsevier’s Publishing Ethics Policy. In particular, the following is fulfilled:

• The work described has not been published previously, nor is it under consideration for publication elsewhere.
• The article's publication is approved by all authors and tacitly or explicitly by the responsible authorities where the work was carried out.
• If accepted, the article will not be published elsewhere in the same form, in English or in any other language, including electronically, without the written consent of the copyright-holder.
• All sources used are properly disclosed.
• All authors have been personally and actively involved in substantial work leading to the paper and will take public responsibility for its content.
• This study did not require the approval of the Ethical Committee for the Care and Use of Experimental Animals of the Free University of Bolzano.

## CRediT authorship contribution statement

**Letizia Debertolis:** Writing – review & editing, Writing – original draft, Visualization, Methodology, Data curation. **Louis Holighaus:** Writing – review & editing, Writing – original draft. **Matthias Gauly:** Writing – review & editing, Conceptualization. **Thomas Zanon:** Writing – review & editing, Conceptualization.

## Declaration of competing interest

The authors declare that they have no known competing financial interests or personal relationships that could have appeared to influence the work reported in this paper.

## Appendix Supplementary materials

Image, application 1
