# Supplementary material for: Review: Compliance standards for dairy cattle welfare in European countries
Source: Vet Anim Sci. 2026 Jan 30;31:100587. doi: 10.1016/j.vas.2026.100587 (PMC12891883; doi:10.1016/j.vas.2026.100587)
Supplement: Supplementary file 1 [file mmc1.docx]

Supplementary material

Modified PRISMA 2020 Flow Diagram

**Identification of CFs via databases and other methods**

CFs removed *before screening*:

Duplicate CFs removed (n = 12)

Non animal welfare-related CFs and other cataloguing errors (n = 10)

CFs identified from:

Google desk search (n = 55)

Database search, citation searching and snowball effect (n = 34)

On indication by person or institution, after direct contact (n= 27)

**Identification**

CFs excluded:

Geographical origin outside Europe (n = 17)

Dairy cattle not addressed as subject matter (n = 9)

**Screening**

CFs assessed for eligibility

(n = 94)

Eligible CFs

(n = 68)

Prioritization process: priority to

1. CFs of which text and regulations are easier to access
2. CFs from regions where dairy sector is most important
3. CFs from regions with longer tradition of animal welfare attention

**Prioritization**

CFs included for overview

(n = 8)

CFs included for comparison

(n = 9)

**Included**

Source: Page et al., 2021 DOI:10.1136/bmj.n71 modified
